# Perceived Social Support and Depressive Symptoms in AYA Cancer Patients: A Mediation Analysis of Hope Agency and Pathways

**DOI:** 10.3390/children13030429

**Published:** 2026-03-20

**Authors:** Julie N. Germann, Colter D. Ray, Holly Rushing, Megan Rittenberg

**Affiliations:** 1 Children’s Health, Dallas, TX 75235, USA; 2 UT Southwestern Medical Center, Dallas, TX 75390, USA; 3 Department of Communication, The University of Tampa, Tampa, FL 33606, USA

**Keywords:** adolescent/young adult (AYA), cancer, hope, agency, pathways, social support, perceived social support, depression, gender

## Abstract

**Highlights:**

**What are the main findings?**

- The agency component of hope mediates the relationship between perceived social support (PSS) and depressive symptoms (D) in adolescent/young adult (AYA) women with cancer.
- The pathways component of hope did not mediate the relationship between PSS and D in AYAs with cancer.

**What are the implications of the main findings?**

- Given the significantly higher prevalence of depression in AYA women with cancer as compared to men, interventions to enhance agency are an important adjunct to interventions to promote PSS and, in turn, reduce D in AYA women with cancer.
- Future studies, including qualitative studies, should strive to elucidate the nature of social support’s influence on hope (both agency and pathways), such as whether specific types of social support (emotional, esteem, informational, network, and tangible) are particularly related to hope or agency, or whether AYA women’s unique social connections or specific factors of social support are most influential on agency.

**Abstract:**

Background/Objectives: Adolescent/young adult (AYA) cancer patients experience a variety of challenges, including higher rates of depressive symptoms (D) than healthy controls. Although various forms of social support (SS) seem to be a protective factor against D, AYAs also experience significant disruptions to SS. Snyder’s hope theory involves two interrelated components: agency (the belief in one’s ability to initiate or sustain action toward a desired goal) and pathways (the belief in one’s ability to produce workable routes to desired goals). Because hope, particularly the agency component, has been linked to both SS and D, it may mediate the relationship between the two. Methods: An online questionnaire was completed by AYA cancer patients (*N* = 205) in which they self-reported levels of perceived social support (PSS), hope, and D, among other scales and demographic variables. A parallel mediation model was conducted to determine if either or both agency and pathways explain the relationship between PSS and D. Results: Agency mediated the relationship between PSS and D in AYA women with cancer, but not men. Pathways did not mediate the relationship between PSS and D. Conclusions: Given the significantly higher prevalence of depression in women with cancer as compared to men, interventions to enhance agency are an important adjunct to methods to promote PSS in AYA women with cancer.

## 1. Introduction

Adolescent/young adult (AYA) survival rates have not advanced to the extent of older adult or pediatric cohorts [1]. This age group (15–39 years old [2]) also comes with a unique set of challenges, such as disruptions to developmental accomplishments, formation of one’s self-concept, and interference with crucial peer interaction [3]. Additionally, AYAs with cancer are at an increased risk of mental health issues such as depressive symptoms [4]. A 2023 meta-analysis found that approximately one in four AYA cancer survivors has experienced depression [4], with additional studies reporting that between 25 and 39% of AYA cancer patients experience depressive symptoms [5,6]. Furthermore, a 2022 study of Japanese AYA cancer patients found that individuals with cancer were three times more likely to experience major depressive disorder (MDD) than a control group of healthy individuals [7]. Intervention is needed to promote positive mental health amongst AYA cancer patients.

### 1.1. Social Support

Social support, broadly defined as “a network of family, friends, neighbors, and community members that is available in times of need to give psychological, physical, and financial help” [8] can act as a protective factor against depressive symptoms [9,10,11,12]. Many studies have found high levels of SS to correlate negatively with depression, and a recent systematic review showed that 89% of existing studies on SS and depression report a notable negative association between the two [13]. Conversely, a recent study [14] found higher total perceived social support (PSS) scores to be correlated with both higher anxiety and depression scores: The study authors’ review of these findings with a youth advisory panel suggested that higher PSS may come with a feeling of duty to protect supporters from the burden of the cancer patient’s emotional pain. Accordingly, participants described having to “be strong” and not actively seeking emotional support even from those who were identified as being available for support.

Despite the complexities of seeking SS, some qualitative studies have reported that AYAs identify SS as a primary strategy for coping with cancer [15,16], including specific supporter behaviors that AYAs find helpful (i.e., presence, distraction, positive attitude, maintaining AYA autonomy, communication, and advocacy [16]). Even though SS provided in any context can be helpful, its impact differs based on source, with SS provided by same-aged peers being especially important for AYA cancer patients [17,18]. However, a cancer diagnosis can cause disruptions in opportunities to interact with peers during treatment (i.e., inability to attend school/work, isolation restrictions, etc.), generally leading to a decrease in peer support systems [19]. Recent research suggests that three out of five cancer patients have experienced nonsupport (i.e., not receiving expected support from close others), which can lead to greater depressive symptoms, worsened mental and physical health, and greater feelings of loneliness [20]. Considering the importance of SS as a protective factor against D despite barriers, a better understanding of the link between SS and D is needed.

### 1.2. Hope

Hope may be a mechanism that explains the previously established links between SS and D in AYAs with cancer. Hope has been described as an “overall perception that one’s goals can be met” [21] (p. 400) and involves two interrelated components: agency and pathways. Agency involves the belief in one’s ability to initiate or sustain action toward a desired goal, whereas pathways involve the belief in one’s ability to produce workable routes to desired goals [22]. In reviewing the available literature, hope was found consistently to be negatively associated with depression and anxiety and positively associated with health, quality of life, self-esteem, adjustment and coping, and social support in adult cancer patients undergoing treatment [23]. In children and adolescents, changes in hope partially mediated the effects of depression and anxiety on quality of life [24]. Given hope’s positive relationship with social support and inverse relationship with depression, it may explain the relationship between SS and D. Because hope is modifiable through the use of solution-focused, narrative, and cognitive-behavioral interventions [25,26] as well as brief skills-based resilience interventions [27], it may be a useful focus of intervention to impact both SS and D.

Although hope has often been examined as a total construct, more recent studies in healthy populations have begun to examine the differential impact of its two components: agency and pathways. In adult populations, agency has been related to negative affect [28]. Similarly, in college student samples, agency emerged as a unique actor in adjustment. Specifically, agency was a predictor of anxiety and depression [29] and suicide risk [30], as well as a mediator of psychological maladjustment [31]. In children and adolescents with cancer, agency was predictive of changes in symptoms of depression and anxiety [32]. In none of these studies was the specific pathways aspect of hope significant. Although these findings occurred in a population of AYAs (college students; [29,30,31]) and a small group of adolescent cancer patients [32], none of these addressed the role of agency and pathways in a population that includes both the full age range of AYAs and the unique cancer context.

### 1.3. The Present Study

This study aims to fill several gaps in the AYA cancer coping literature. First, since SS has been identified as an important protective factor against D [13] despite significant barriers to AYAs receiving SS in the cancer context [19], a better understanding of the link between SS and D is needed. Because hope has been separately associated with both SS and D [23], it may be an important explanatory factor, but hope has not yet been explored in this way. Next, studies within the hope field have started to focus on exploring the differential influence of agency and pathways, with agency beginning to emerge as more influential than pathways on a variety of outcome variables when looking at combined samples of males and females [28,29,30,31,32]. However, as the hope literature is emerging and has not been studied specifically in AYAs with cancer, we will evaluate both agency and pathways.

We propose that both components of hope (agency and pathways) mediate the relationship between PSS and D in AYAs with cancer. This is stated formally as a hypothesis:

**H1.**  *The relationship between perceived social support (PSS) and depression (D) is mediated by (H1a) agency and (H1b) pathways.*

## 2. Materials and Methods

### 2.1. Setting

This study analyzed a subset of data from a larger study focused on AYA cancer patients’ perceived barriers to seeking support and their reactions to instances of nonsupport and the acceptability of these instances when people expected to provide support chose not to do so [20,33,34]. These prior analyses did not explore the data collected regarding hope or depression, which is the focus of this study. Data collection was conducted entirely online through questionnaires created using the survey hosting platform Qualtrics, Provo, UT, USA. Participants were recruited by contacting the company Prolific Academic, London, UK, to specifically locate and recruit young adult cancer patients, and this data collection occurred throughout May and June of 2022.

### 2.2. Participants

Participants were 205 young adult cancer patients ranging in age from 18 to 39 years (*M* = 28.52, *SD* = 5.35) who reported that their initial diagnosis had occurred between 0 and 23 years ago (*M* = 6.50, *SD* = 4.56). Participants were from 22 countries, with approximately 75% of the participants living in the United States, the United Kingdom, Poland, or South Africa. Demographic information for this study’s sample has been previously reported [20] and is reproduced here for completeness in Table 1.

### 2.3. Procedure

Participation consisted of completing two questionnaires that were distributed three months apart; however, the data for this study’s analyses were collected during the first of the two questionnaires (i.e., Time 1). After providing informed consent, participants provided demographic information and responded to open-ended questions about barriers to seeking support and whether they ever experienced nonsupport (i.e., instances when support was expected but not received). The data used for this study were collected in the second half of the Time 1 questionnaire, which consisted of several scales that measured variables such as perceived social support, hope, and depression. Detailed information on these measures is provided next.

### 2.4. Measures

Demographic information. Participants provided background information that is both typical of social scientific studies (e.g., age, gender identity, and ethnicity) and specific to their cancer journey (e.g., cancer type and stage and treatments completed).

Perceived social support. The Multidimensional Scale of Perceived Social Support (MSPSS; [35]) is a 12-item self-report scale that measures the perceived adequacy of support from family (e.g., “My family really tries to help me”), friends (e.g., “I can count on my friends when things go wrong”), and significant others (e.g., “There is a special person in my life who cares about my feelings”). Response options are provided in a seven-point Likert-style format (1 = Very Strongly Disagree; 7 = Very Strongly Agree). The scale’s developers report between good and excellent internal reliability at the time of the scale’s development (0.81–0.90 for the Family subscale; 0.90–0.94 for the Friends subscale; 0.83–0.98 for the Significant Other subscale; from 0.84 to 0.92 for the scale as a whole; [35]).

Hope. The Adult Hope Scale (AHS; [36]) is a 12-item self-report measure for individuals 15 years and older. Four questions address agency (e.g., “I meet the goals that I set for myself”), four items address pathways (e.g., “I can think of many ways to get the things in life that are most important to me”), and four items are distraction items that are not included when scoring the scale (e.g., “I feel tired most of the time”). Response options are presented as four-point Likert-style scales (1 = Definitely False; 4 = Definitely True). The scale generates three scores: an overall hope score that combines agency and pathways (theoretically ranging from 8 to 64) and individual agency and pathways subscores (each potentially ranging from 4 to 32). Prior research studies that use the AHS [36] have reported good levels of internal reliability (alphas of 0.74–0.84 for the overall scale and 0.63–0.80 for the agency and pathways factors) and factor structure. Concurrent, discriminant, and convergent validity have been supported in research [36].

Depressive symptoms. The Iowa short form of the Center for Epidemiological Studies Depression Scale (CES-D Scale) [37] consists of 11 items (e.g., “I felt sad,” “I felt that everything was an effort”). Participants reported how often they experienced each item over the past seven days, with response options presented in Likert-style scales with the response options of 0 (Hardly Ever or Never), 1 (Some of the Time), or 2 (Much or Most of the Time). Before calculating a summed score, responses for two items were reverse-coded: “I was happy” and “I enjoyed life.” Summed scores on this scale can potentially range from 0 to 22, with higher scores indicating greater self-reported depression. At the time of development, the Iowa short-form version of the CES-D showed adequate internal reliability, with a Cronbach’s alpha score of 0.76 [37].

### 2.5. Data Analysis

Once the final sample for analysis had been identified by removing prospective participants who did not meet our inclusion and exclusion criteria, the authors began data analysis by addressing instances of missing data. Across 205 participants responding to 35 items across the scales measuring perceived social support, hope, and depression, there were only seven instances of missing responses out of 7175 possible responses (i.e., less than 0.01% of data was missing). Given that missing data were exceedingly rare, these seven instances of missing data were addressed by imputing the mean. Next, descriptive statistics were conducted, and internal reliability was assessed for the three scales used to measure our variables of interest. The intercorrelations of the study’s variables, their descriptive statistics, and their internal reliability scores are reported in Table 2.

Hayes’s PROCESS Macro (Model #4) for SPSS (Version 31) was used to test our hypotheses. This specific model allows for a test of parallel mediation and the use of a pairwise contrast of indirect effects to determine if one indirect effect is statistically stronger than the other. In our model, our independent variable (X) was PSS, our dependent variable (Y) was D, and our two mediator variables (arranged in parallel) were the two factors of the Adult Hope Scale: Agency (M_1_) and Pathways (M_2_). When running the model, continuous variables were standardized (i.e., z-scores were used) and mean-centered. This means that the coefficients reported correspond to standardized effects (i.e., beta coefficients). 5000 bootstrap samples were used to estimate confidence intervals for the indirect effects. Lastly, because the age of the participant significantly correlated with perceived social support (*r* = 0.21, *p* = 0.003) and depression (*r* = −0.19, *p* = 0.005), age was included as a covariate in our analyses. Other variables were also tested as potential covariates (e.g., time since diagnosis, cancer staging, and whether the participant had completed primary treatment); however, these variables did not change the pattern of results and were excluded from the analysis for the sake of parsimony.

## 3. Results

### 3.1. Summary of Hypothesis Tests

**Figure 1:**
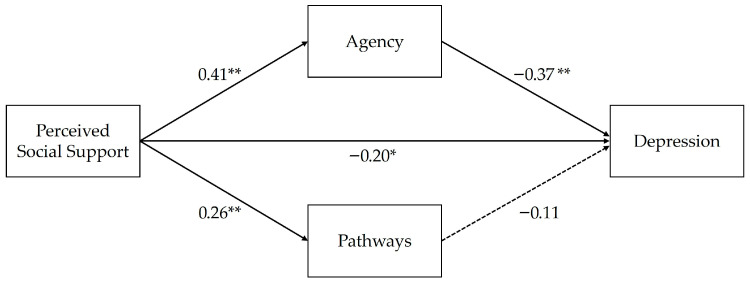
Parallel mediation model testing the indirect effects of hope (agency and pathways). Notes: * *p* < 0.01; ** *p* < 0.001.

We hypothesized that the relationship between PSS and D would be mediated by the agency component of hope (H1a) and the pathways component of hope (H1b). Results showed that the agency component of hope indeed mediates the relationship between PSS and D, thus providing support for H1a. The pathways component of hope, however, was not significant, and H1b was not supported. Furthermore, the indirect effect using agency as a mediator was significantly stronger than the indirect effect observed when using pathways as a mediator. The following subsections report a fuller picture of these results, starting with the direct effect of PSS on D and then reporting results for each hypothesis. Results are presented visually in Figure 1, and detailed results are listed in Table 3.

### 3.2. Unhypothesized Direct Effects

There was a significant direct effect of PSS on D while controlling for age (β = −0.20, SE = 0.06, *p* = 0.002). Although this direct effect was not specifically hypothesized, this result aligns with the findings from numerous prior studies that have shown an inverse relationship between PSS and D (see [13]).

### 3.3. H1a: Mediation via Agency

H1a was supported due to the results showing that agency significantly mediates the relationship between PSS and D (completely standardized indirect effect = −0.18, SE = 0.03, 95% CI [−0.22, −0.09]). There was a significant positive relationship between PSS and agency (β = 0.41, SE = 0.07, *p* < 0.001, 95% CI [0.28, 0.54]) and, in turn, agency was significantly negatively related to D (β = −0.37, SE = 0.08, *p* < 0.001, 95% CI [−0.52, −0.21]).

### 3.4. H1b: Mediation via Pathways

H1b was not supported, as the overall indirect effect using pathways as a mediator was not significant (completely standardized indirect effect = −0.03, SE = 0.02, 95% CI [−0.08, 0.01]). Although the relationship between PSS and pathways was significant and positive (β = 0.26, SE = 0.07, *p* < 0.001, 95% CI [0.12, 0.40]), the association between the pathways component of hope and D was nonsignificant (β = −0.11, SE = 0.07, *p* = 0.136, 95% CI [−0.26, 0.04]). Additionally, a bootstrap pairwise contrast of indirect effects further confirmed the results of H1a and H1b by showing that the indirect effect of agency was significantly stronger than the indirect effect of pathways (contrast = −0.12, 95% CI [−0.22, −0.03]).

### 3.5. Post Hoc Exploratory Analyses: Does Gender Matter?

When considering how hope may mediate the relationship between PSS and D, another factor to consider is the AYA cancer patient’s gender. For example, Chang [28] strongly advocated for more explicit tests of gender differences in the hope components’ relationship to outcomes such as depression, given that adolescent and adult women experience a higher prevalence of depression than men [38], which has also been found within AYA cancer populations [14]. Although agency emerged as the most active ingredient in this study, as it has in prior studies [29,30,31,32], one study did find gender differences, with men scoring higher on both agency and pathways [28].

Prior research has also found differences in the use of coping strategies between genders, with studies showing that women are more likely to use emotion-focused strategies, whereas men are more likely to use problem-focused strategies [39]. This may map onto the agency and pathways components of hope. Specifically, because men often use problem-focused strategies, the pathways component of hope may be a stronger mediator between PSS and D for men. Conversely, the agency component of hope focuses on one’s self-efficacy, which is more closely aligned with the outcomes of emotion-focused support. Given that men are more likely to use problem-focused strategies, we decided to conduct post hoc exploratory analyses to determine if, for men, the relationship between PSS and D would be mediated through the pathways component of hope. Likewise, we also explored if the agency acts as a stronger mediator of the PSS and D relationship for women than it does for men.

To investigate this, an additional six participants were excluded from the exploratory analyses as they reported their gender identity as something other than cisgender man or cisgender woman, and this exploratory analysis specifically tests for differences in indirect effects between men and women. There were not enough third-gender or non-binary participants to conduct a statistical analysis to explore this possibility for those identifying as something other than cisgender.

PROCESS Model #59 (moderated mediation) was used to conduct these post hoc exploratory analyses. The moderator variable was gender identity (scored as 0 = female and 1 = male), and this moderator was tested on each pathway in the original mediation model used to test our hypotheses (i.e., gender was tested as a moderator on the mediation paths X-M_1_, X-M_2_, M_1_-Y, M_2_-Y, and the direct effect X-Y). Because the model includes interaction terms due to testing gender as a moderator, standardized beta coefficients are not appropriate to report. Instead, unstandardized coefficients (b-values) are reported for the moderated mediation models. To facilitate interpretation of these results, all continuous variables were standardized prior to the analysis (i.e., z-scores were used), meaning that the reported coefficients represent effects in standard deviation units. Therefore, each coefficient reflects the expected change in outcome (in standard deviation units) associated with a one standard deviation change in the preceding variable.

Overall, the results of this exploratory analysis act as an important caveat to our hypothesis tests. Results showed that the agency component of hope mediates the relationship between PSS and D, but only for women (indirect effect = −0.28, SE = 0.06, 95% CI [−0.40, −0.16]) and not for men (indirect effect = −0.05, SE = 0.04, 95% CI [−0.12, 0.04]). This is further supported by a significant result for the index of moderated mediation for the indirect effect using agency as the mediator (index of moderated mediation = 0.23, SE = 0.07, 95% CI [0.10, 0.38]), demonstrating that agency was a significant mediator for women but not for men. Although this was not statistically significant for men, the relatively wide confidence interval suggests that the analysis had limited statistical power to detect a significant effect within the male subgroup, given that only 77 participants were men.

Even in exploring the possibility that pathways are a mediator specifically for men but not women, the results were nonsignificant. That is, the indirect effect using pathways is not significant, regardless of whether it is tested across genders (as it was in H1b, which was not supported) or when specifically looking at the results for men or women separately. One promising aspect of this exploratory analysis was that there was a significant indirect effect for men (indirect effect = −0.07, SE = 0.04, 95% CI [−0.16, −0.003]) and not for women (indirect effect = 0.01, SE = 0.04, 95% CI [−0.08, 0.09]); however, the index of moderated mediation was nonsignificant, which leads to the determination that the overall indirect effect using pathways as a mediator does not reliably differ based on gender (index of moderated mediation = −0.08, SE = 0.06, 95% CI [−0.21, 0.03]).

In synthesis, H1a was supported, suggesting that agency mediates the relationship between PSS and D. However, our exploratory analyses found that this indirect effect only occurs for AYA women patients and not for AYA men patients. Alternatively, using pathways as a mediator (H1b) yielded nonsignificant results, in general. When exploring if gender moderated this indirect effect, the indirect effect became significant for men only, but the difference between men and women was not consistent, meaning that we cannot claim a gender difference exists.

## 4. Discussion

### 4.1. Agency as a Mediator of Perceived Social Support and Depressive Symptoms in AYA Women with Cancer

This study attempted to fill several gaps in the AYA cancer literature. This study was the first to examine each of the following in AYAs with cancer: (1) hope as a mediator of the relationship between PSS and D, (2) specifically focusing on the differential impact of agency and pathways, and (3) while also exploring gender differences in agency and pathways. Our results indicated that agency was a partial mediator between PSS and D, but for women only, while pathways were not a significant mediator at all, partially confirming hypothesis H1a. In other words, for AYA women with cancer, agency may help explain the connection between PSS and D, which suggests that part of PSS’s impact on D is due to women’s use of agentic thinking. These findings align with prior research indicating that agency may be the more active ingredient associated with adjustment [28,29,30,31,32].

Our findings also bolster the hope literature with the finding that agency may be affected by the AYA gender. Because women are more likely to rely on emotion-focused strategies [39,40,41,42], and because the agency component of hope focuses on one’s self-efficacy, which is more closely aligned with the outcomes of emotion-focused support, we hypothesized that agency would be a mediator for AYA women in our study, which was confirmed. Conversely, because men report greater use of problem-solving strategies [39,41], which may map onto pathways, we hypothesized that pathways would be a mediator for AYA men in our study; however, this hypothesis was not supported. This extends findings from a study of middle-aged men and women, showing that the association between agency and depressive symptoms was stronger in women than in men [28]; interestingly, Chang [28] also found that the relationship between agency and problem-solving (as a separate, measured construct) was stronger in women than in men. This implies that problem-solving is not synonymous with or mapped directly onto pathways; rather, Chang suggested that when women engage in problem-solving activities, they may rely more heavily on thoughts related to their sense of agency than men do. In our sample, perhaps AYA women with cancer who perceive high social support have fewer depressive symptoms because they utilize more agentic thinking.

This is an important exploratory finding considering the higher prevalence of depression in women, not only in the general population [38] but in cancer populations specifically [14]. In our sample, the direct effect between PSS and D was not moderated by gender, underscoring the general importance of SS to coping with cancer for both AYA men and women; however, the fact that in AYA women this is partially due to agency highlights the need to better understand this mechanism. Perhaps AYA women who feel they have a high amount of social support feel more energy or motivation to engage in coping strategies and manage the stress of a cancer diagnosis, thereby experiencing fewer depressive symptoms. Perhaps these women feel more energized by their support networks. Perhaps those with higher agency are more proactive and effective at accessing and utilizing the social support they feel they have and, therefore, ameliorate the impact of cancer on their mood. Perhaps they feel that with strong social support (in any of its possible forms), they can delegate tasks, utilize resources, share feelings, or access any other type of support they need so that they can utilize their remaining energy to more effectively manage their mood and cope with cancer. Alternatively, perhaps they feel more responsibility and motivation to manage coping and distress so as not to burden their support networks. All of these reasons are based solely on conjecture but would be important foci for future research and would likely benefit from not only quantitative but also qualitative studies to elucidate and explain this connection (see Future Directions).

Until we have a better understanding of the nuances of how agency impacts the connection between PSS and D, these findings nevertheless indicate that for AYA women with cancer to address D, it may be important not only to improve PSS (as it is with AYA men) but also to focus on agency. Women in the AYA age range (ages 18–39 in this study) are in a phase of life where they are establishing their identity, careers, relationships, and possibly starting families [43]. Women are balancing a number of roles and duties, including managing the home, physical health, and behavioral health of themselves and their loved ones [44,45]. These responsibilities persist, even when faced with a cancer diagnosis and treatment. Agency, in conjunction with PSS, may be crucial in helping these AYA women manage all these tasks while meeting their general goals and preventing depressive symptoms.

### 4.2. Clinical Implications

These findings have several clinical implications. The direct effect of PSS on D reinforces the importance of assessing AYA perceptions of their social support [46] and intervening to address barriers accordingly (specific SS-enhancing recommendations have been detailed elsewhere [34]). However, the mediating effect of agency in AYA women with cancer points to the importance of assessment and interventions to increase agency, in conjunction with interventions focused on SS. It is possible that individuals’ agency and pathways are derived not only from their own perceived capability but also from external supports, such as family and peers [47]. While Bernardo [47] proposed this modification to hope theory as a better fit across cultures, which may have a more collectivist orientation, deriving hope (and its components) from social supports may also be especially important in the cancer context, particularly for AYAs who experience significant disruptions to normative developmental social connections during and after treatment.

Several hope-focused interventions have been developed and evaluated [26,27], while a variety of validated psychotherapeutic strategies (solution-focused, narrative, and cognitive-behavioral interventions) have also impacted hope [25]. Interventions focusing on hope have not traditionally focused exclusively on one component over another; however, clinical experience provides some useful suggestions for enhancing agency. An agency-specific intervention, focused on enhancing a person’s perceived capability to begin and maintain movement toward a desired goal, might include identifying barriers to initiating action, identifying previously used successful strategies for completing challenging tasks, using cognitive strategies to reinforce an individual’s awareness of their successes (enhancing self-efficacy), using positive visualization to envision past and future processes of pursuing a goal [25], and addressing contributors to low mental and physical energy (e.g., side effects, sleep hygiene, poor nutrition). These interventions, whether focused on hope generally or agency specifically, could be provided in conjunction with the aforementioned strategies to increase social support, thereby working reciprocally to reduce depressive symptoms. While our study identified the role of agency for AYA women only, it is nevertheless important to assess individual differences with each patient; accordingly, some men may benefit from agency-focused interventions, while they may be irrelevant for some women.

### 4.3. Limitations and Future Directions

This study has several limitations, which can be addressed with future research. These analyses were cross-sectional, which limits conclusions about causality. Additional longitudinal tests of this model must occur to establish causal relationships among PSS, hope agency and pathways, and D. However, the use of cross-sectional data to test mediation is justifiable when it can be argued that the independent variable precedes the mediator variables in time in terms of the order that someone would experience the variables in a real-life (non-research) situation (i.e., the Hyman-Tate conceptual timing criteria; [48]).

Another limitation of this study is the focus specifically on a measure of PSS, which does not provide details on specific forms of support or actual support received. Future studies, including longitudinal quantitative as well as qualitative studies, could help determine if certain forms of support (emotional, esteem, informational, network, tangible) are likely to correlate with specific aspects of hope (agency vs. pathways). For example, pathways may be more strongly associated with problem-focused forms of support, whereas emotion-focused forms of support may correlate more strongly with the agency component of hope. Exploring this in future studies would provide a better understanding of how SS generally impacts hope and its components, and in turn, D. Additional analyses were conducted to test whether different sources of support (i.e., the three dimensions of the scale used to measure PSS) affected the findings. Regardless of whether AYA cancer patients were perceiving support from family members, friends, or significant others, the same pattern of results occurred: PPS influenced D through hope agency but not hope pathways. Although future researchers could continue to explore the potential effects of the source of support, the more promising future direction is to explore this model in terms of emotion-focused versus problem-focused forms of support.

Our sample included individuals who were, as a cohort, experiencing generally low levels of D, and while participants did report cancer stage/risk and treatment type, it is unknown what their specific prognosis or current health status was at the time they completed the questionnaires. Future studies should specifically test the associations between SS, hope, and D in AYA populations that differ in factors that could affect these constructs, such as cancer diagnosis severity and prognosis or depression severity (for example, those who are clinically depressed versus those who are not).

Additionally, the model itself does not involve any variables specific to the cancer experience. Instead, it is a model that uses common variables in psychosocial research and was tested in this study within the AYA cancer population. This suggests that the model may not be specific to only AYA cancer patients and may also be applicable to other life stressors experienced by AYAs, regardless of whether they are cancer patients (e.g., the death of a family member or experiencing the end of a romantic relationship). Future researchers ought to test this model in other domains beyond cancer to better understand the boundaries of the model and whether it is specific to the AYA cancer experience.

While our sample included a population that was diverse in terms of geography, education, income, relationship status, cancer type, cancer stage/risk, and treatment type, our sample was composed primarily of White participants (78%), and, for the gender-as-moderator analysis, we excluded non-cisgender participants due to small numbers that precluded reasonable statistical comparisons. Future studies should make efforts to increase inclusion and participation of ethnically diverse and non-cisgender participants, as these participants could experience significantly different social support and hope.

## 5. Conclusions

This study is the first to examine the specific roles of agency and pathways (hope) as mediators between perceived social support (PSS) and depression (D) symptoms in AYAs with cancer according to gender. Our findings that agency mediates the link between PSS and D in AYA women with cancer reinforce the value of examining the separate hope components as well as their differential expression by gender. Because women experience higher levels of depression, both in general populations and in cancer populations, it is important to maximize interventions that can address multiple factors impacting depression. Although social support is an important protective factor for AYA men and women with cancer, social support’s positive impact appears to be partially due to the specific hope component of agency, thereby pointing to the importance of agency-enhancing interventions in conjunction with social support interventions. Future research should continue to test this model, specifically through longitudinal repeated measures designs to test the temporality of the mediation model and to establish causality. Longitudinal studies would also allow for tracking how changes in PSS and hope (agency and pathways) may affect D as AYA cancer patients progress from initial diagnosis to primary treatment and into survivorship.

## Figures and Tables

**Table 1:** Young adult cancer patients’ demographic information and cancer experiences (*N* = 205).

	*n* (%)
Race/Ethnicity	
White	159 (77.9%)
Black/African American	25 (12.3%)
Asian	13 (6.4%)
Latinx/Hispanic	14 (5.4%)
Native American/Alaskan Native	2 (1.0%)
Native Hawaiian or Pacific Islander	2 (1.0%)
Prefer Not to Answer	1 (0.5%)
Gender	
Woman	122 (59.5%)
Man	77 (37.6%)
Nonbinary/3rd Gender	4 (2.0%)
Trans Man	1 (0.5%)
No Response	1 (0.5%)
Sexual Orientation	
Straight	155 (75.6%)
Bisexual	33 (16.1%)
Gay/Lesbian	10 (4.9%)
Pansexual	3 (1.5%)
Asexual	2 (1.0%)
Queer	1 (0.5%)
No response	1 (0.5%)
Education ^1^	
Did not complete high school	3 (1.5%)
High school or equivalent	21 (10.2%)
Technical, trade, or vocational school	5 (2.4%)
Some college but no degree	31 (15.1%)
Associate’s degree	8 (3.9%)
Bachelor’s degree	85 (41.7%)
Master’s degree	40 (19.5%)
Doctoral degree (PhD)	4 (2.0%)
Professional degree (e.g., JD, MD, DDS)	7 (3.4%)
Prefer Not to Answer	1 (0.5%)
Geographic Location ^2^	
Europe	117 (57.1%)
North America	66 (32.2%)
Africa	17 (8.3%)
Oceania	5 (2.4%)
Household Income ^3^	
$0	3 (1.5%)
$1–$9999	19 (9.3%)
$10,000–$24,999	39 (19.0%)
$25,000–$49,999	56 (27.3%)
$50,000–$74,999	33 (16.1%)
$75,000–$99,999	26 (12.7%)
$100,000–$149,999	14 (6.8%)
$150,000 or more	7 (3.4%)
Prefer Not to Answer/No Answer/Unsure	8 (3.9%)
Romantic Relationship Status ^4^	
Married	47 (22.9%)
Single/Not in a Committed Relationship	72 (35.1%)
Committed Dating Relationship	71 (34.6%)
Engaged	13 (6.3%)
Prefer Not to Answer/No Answer	2 (1.0%)
Cancer Type	
Lymphoma	39 (19.0%)
Breast	26 (12.7%)
Thyroid	20 (9.8%)
Testicular	19 (9.3%)
Leukemia	18 (8.8%)
Ovarian	9 (4.4%)
Lung	7 (3.4%)
Bone	6 (2.9%)
Other ^5^	34 (16.6%)
Cancer Stage or Risk	
Stage 0	3 (1.5%)
Stage 1	40 (19.6%)
Stage 2	61 (29.9%)
Stage 3	36 (17.2%)
Stage 4/Metastatic	26 (12.7%)
Low Risk/Chronic Phase	12 (5.9%)
Medium Risk	2 (1.0%)
High Risk	10 (4.9%)
Not Staged or Not Recalled	9 (4.5%)
No Response	6 (2.9%)
Treatment(s)	
Chemotherapy	114 (55.6%)
Radiation Therapy	52 (25.5%)
Surgery	122 (59.8%)
Immunotherapy	5 (2.5%)
Stem Cell Transplant	4 (2.0%)
Hormone Therapy	4 (2.0%)
Bone Marrow Transplant	2 (1.0%)
Tyrosine Kinase Inhibitor Target Therapy	2 (1.0%)
No Treatment Prescribed Yet	1 (0.5%)
Primary Treatment Completed?	
Yes	178 (86.8%)
No	27 (13.2%)

Notes. Percentages for each attribute may not equal 100% exactly due to rounding errors. Ethnicity/race, cancer sites, and cancer treatments sum to greater than 100% because some participants identified as two or more races or ethnicities or reported multiple cancer sites or treatments. ^1^ Highest level of education completed unless otherwise noted. ^2^ In total, participants reported living in 22 different countries. Although the majority of participants lived in Europe, the most frequently reported place that participants lived was the United States (*n* = 58). Participants living in the United States reported living throughout 25 different states. ^3^ Income reported in $USD. ^4^ No participants reported being divorced, separated, or widowed. ^5^ Fourteen additional cancer types were reported by five or fewer participants, and these were categorized as “Other” for the sake of brevity.

**Table 2:** Zero-order correlations and descriptive statistics (*N* = 205).

Variable	1.	2.	3.	4.	5.	*M*	*SD*	ω	Observed Range
1. Perceived Social Support	—	0.41 **	0.26 **	−0.41 **	0.21 *	5.28	1.15	0.87	1.67–7.00
2. Hope (Agency)		—	0.64 **	−0.53 **	0.05	11.01	2.41	0.77	4.00–16.00
3. Hope (Pathways)			—	−0.41 **	0.06	11.74	2.14	0.78	5.00–16.00
4. Depression				—	−0.19 *	8.60	5.18	0.88	0.00–22.00
5. AYA Patient Age					—	28.52	5.35	—	18.00–39.00

Notes. * *p* < 0.01 (two-tailed). ** *p* < 0.001 (two-tailed). ω = the internal reliability statistic McDonald’s omega.

**Table 3:** Standardized regression coefficients and indirect effects of the parallel mediation model testing H1a and H1b (*N* = 205).

Path	β	SE	95% CI
Outcome: Hope (Agency)			
Perceived Social Support → Agency	0.45	0.07	[0.32, 0.59]
Age → Agency	−0.01	0.01	[−0.03, 0.02]
Outcome: Hope (Pathways)			
Perceived Social Support → Pathways	0.29	0.07	[0.15, 0.44]
Age → Pathways	−0.00	0.01	[−0.03, 0.02]
Outcome: Depression			
Perceived Social Support → Depression (direct effect)	−0.21	0.07	[−0.34, −0.07]
Hope (Agency) → Depression	−0.37	0.08	[−0.52, −0.21]
Hope (Pathways) → Depression	−0.10	0.08	[−0.25, 0.05]
Age → Depression	−0.02	0.01	[−0.05, −0.00]
Total Effect			
Perceived Social Support → Depression	−0.34	0.07	[−0.48, −0.21]
Indirect Effects (Bootstrapped)			
Via Hope (Agency)	−0.28	0.06	[−0.40, −0.16]
Via Hope (Pathways)	0.01	0.04	[−0.08, 0.09]
Total Indirect Effect	−0.27	0.07	[−0.41, −0.15]

## Data Availability

The raw data supporting the conclusions of this article will be made available by the authors on request. Sharing the data could potentially compromise the privacy of the participants, as stipulated in the ethics approval. Requests to access the datasets may be considered on a case-by-case basis, subject to review and approval by the authors and the appropriate ethics board.

## Abbreviations

The following abbreviations are used in this manuscript:

AYAs	Adolescents/Young Adults
D	Depressive symptoms
SS	Social Support
PSS	Perceived Social Support
MSPSS	Multidimensional Scale of Perceived Social Support
AHS	Adult Hope Scale
CES-D	Center for Epidemiological Studies Depression Scale
SPSS	Statistical Package for the Social Sciences
